# Reply: Low-Grade Inflammation: A Familiar Factor in Cardiovascular Diseases

**DOI:** 10.1016/j.jacbts.2023.09.009

**Published:** 2023-11-27

**Authors:** Tomohisa Sakaue, Hironori Izutani

**Affiliations:** a Department of Cardiovascular Surgery, Ehime University Graduate School of Medicine, Ehime, Japan

We thank Dr Alur for his valuable suggestions regarding our recent findings.^1^ As Dr Alur pointed out, in relation to the connection between low-grade inflammation (LGI) and cardiovascular diseases, several findings have been reported. A previous randomized study potentially suggests that the treatment of periodontitis reduces the expression levels of inflammation markers and blood pressure in patients with hypertension.^2^ LGI is also suggested as a risk factor for cardiovascular events in patients with type 2 diabetes.^3^ Although evidence regarding the roles of LGI in the pathological progression of major cardiac events has accumulated, critical evidence on whether LGI promotes structural valve deterioration with calcification is poorly documented. Given that a histological finding suggesting LGI pathology in explanted bioprosthetic valves with calcification was reported,^4^ LGI may potentially accelerate the calcification of implanted bioprosthetic valves through the infiltration of inflammatory proteins, including fibrinogen and circulating macrophages, into the interstitium of the valve. Future clinical and basic studies focusing on the role of LGI in the valve interstitium and other tissues, including the oral cavity and gut, will be crucial for uncovering the entire mechanism of structural valve deterioration and advancing the treatment of patients with valvular heart disease in cardiovascular surgery.
